# Disparate Associations of HLA Class I Markers with HIV-1 Acquisition and Control of Viremia in an African Population

**DOI:** 10.1371/journal.pone.0023469

**Published:** 2011-08-17

**Authors:** Wei Song, Dongning He, Ilene Brill, Rakhi Malhotra, Joseph Mulenga, Susan Allen, Eric Hunter, Jianming Tang, Richard A. Kaslow

**Affiliations:** 1 Department of Epidemiology, University of Alabama at Birmingham, Birmingham, Alabama, United States of America; 2 Department of Medicine, University of Alabama at Birmingham, Birmingham, Alabama, United States of America; 3 Rwanda-Zambia HIV-1 Research Group, Lusaka, Zambia; 4 Department of Pathology and Laboratory Medicine, Emory University, Atlanta, Georgia, United States of America; 5 Vaccine Research Center, Emory University, Atlanta, Georgia, United States of America; Karolinska Institutet, Sweden

## Abstract

**Background:**

Acquisition of human immunodeficiency virus type 1 (HIV-1) infection is mediated by a combination of characteristics of the infectious and the susceptible member of a transmission pair, including human behavioral and genetic factors, as well as viral fitness and tropism. Here we report on the impact of established and potential new HLA class I determinants of heterosexual HIV-1 acquisition in the HIV-1-exposed seronegative (HESN) partners of serodiscordant Zambian couples.

**Methodology/Principal Findings:**

We assessed the relationships of behavioral and clinically documented risk factors, index partner viral load, and host genetic markers to HIV-1 transmission among 568 cohabiting couples followed for at least nine months. We genotyped subjects for three classical HLA class I genes known to influence immune control of HIV-1 infection. From 1995 to December 2006, 240 HESNs seroconverted and 328 remained seronegative. In Cox proportional hazards models, HLA-A*68:02 and the B*42-C*17 haplotype in HESN partners were significantly and independently associated with faster HIV-1 acquisition (relative hazards = 1.57 and 1.55; *p* = 0.007 and 0.013, respectively) after controlling for other previously established contributing factors in the index partner (viral load and specific class I alleles), in the HESN partner (age, gender), or in the couple (behavioral and clinical risk score). Few if any previously implicated class I markers were associated here with the rate of acquiring infection.

**Conclusions/Significance:**

A few HLA class I markers showed modest effects on acquisition of HIV-1 subtype C infection in HESN partners of discordant Zambian couples. However, the striking disparity between those few markers and the more numerous, different markers found to determine HIV-1 disease course makes it highly unlikely that, whatever the influence of class I variation on the rate of infection, the mechanism mediating that phenomenon is identical to that involved in disease control.

## Introduction

In sub-Saharan Africa, heterosexual exposure accounts for much of the spread of human immunodeficiency virus type 1 (HIV-1) infection, especially among HIV-1 discordant couples [1]. Epidemiological evidence suggests that acquisition of HIV-1 infection is often mediated by risk behaviors (exposure), viral characteristics (subtypes and co-receptor tropism), coinfection with other pathogens [2], and host genetic variations that mediate innate and adaptive immune responses [3], [4]. Longitudinal studies in Lusaka, Zambia have attested to the role of several factors in the index partner as determinants of HIV-1 transmission within HIV-1 discordant couples, namely HIV-1 viral load (VL) [5], [6], genital ulcer or inflammation (GUI) [6], [7], and certain polymorphisms in HLA class I genes (HLA A*36, B*57 and C*18) [6]. For the susceptible HIV-1 exposed seronegative (HESN) heterosexual partners, major involvement of human genetic variants other than those in *CCR5* receptor/ligand system have been less firmly established [3], [8]. Few investigations have included relatively large numbers of paired index and susceptible partners, followed them for long enough, and included sufficient detail to allow persuasive tests of immunogenetic hypotheses.

Human leukocyte antigen (HLA) class I genes in the major histocompatibility complex (MHC) are important determinants of effective immune surveillance. Their allelic variants have been associated with various outcomes in the natural course of HIV-1 infection, including viremia and disease progression (time to manifest immunodeficiency after infection) [9], [10], [11], [12], [13], [14], [15]. Favorable HLA alleles like HLA-B*57 and B*27 have strong and durable impact on both early and late outcomes including set-point VL [6], [16], [17], [18], [19], [20], [21], [22], and they appear to reduce or delay viral transmission by suppressing viremia in an infected potential transmitter (e.g., a sexual partner) [6],[21]. In contrast, evidence that HLA variants influence acquisition in HESNs is less convincing; associations reported for various class I alleles (A*23, A*68:02, A*74 and B*18) have been less consistent in studies of mother-infant pairs [23], [24], commercial sex workers [25], [26], [27] and other high-risk groups [28]. Occasional detection of HIV-1-specific cytotoxic T-lymphocytes (CTLs) in genital mucosa of HESNs [29] has been taken to imply a role for HLA class I alleles in preventing viruses from disseminating and inducing systemic antibody responses, but multiple studies have not shown enough consistency to establish unequivocally the involvement of HLA class I polymorphisms in variable susceptibility to HIV infection [30]. Here in a large cohort of serodiscordant Zambian couples we further document influences of HLA class I alleles on the rate of HIV-1 acquisition that are disparate from those that control viremia.

## Results

### Overall characteristics of HIV-1 discordant Zambian couples in this study

**Figure 1:**
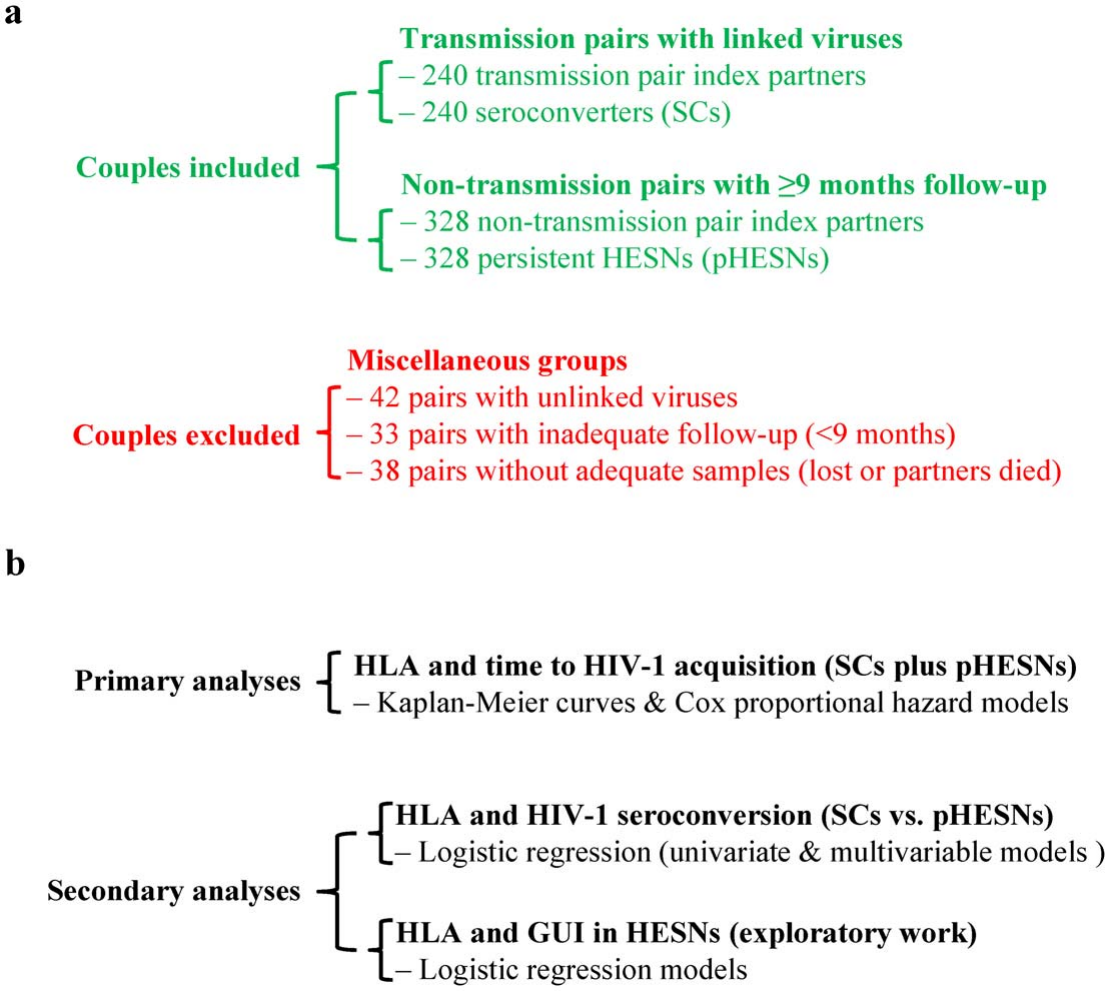
Selection of Zambian subjects for this study (Panel a) and summary of statistical strategies (Panel b). In all, 568 couples (240 transmission pairs and 328 non-transmission pairs) were eligible (see Table 1). Non-genetic factors included age of HESN, sex, direction of transmission (M to F or F to M), genital ulcer/inflammation (GUI), and viral load (VL).

We analyzed 568 HIV-1 serodiscordant couples with complete HLA class I genotyping as well as adequate follow-up between 1995 and 2006 (**Figure 1**). These couples included 240 who seroconverted (SCs) with viruses (predominantly subtype C) closely linked to those found in their index partners and 328 susceptibles who were persistently HESN (pHESN) during quarterly follow-up visits. The pHESNs differed from SCs in sex ratio (*p* = 0.0002), age (*p* = 0.014 for men and *p* = 0.004 for women), time of follow-up from enrollment (*p*<0.0001), and behavioral and clinical risk scores (*p*<0.0001) (**Table 1**). Mean VL was higher in male than in female index partners (*p*<0.0001). Other characteristics, including enrollment date, cohabitation time and age difference between the man and the woman within a partnership, were highly comparable between SCs and pHESNs (data not shown). Therefore, VL of index partner, age and gender of HESN, and risk score were included as covariates in primary analyses of HIV-1 acquisition.

**Table 1:** Selected characteristics of 568 Zambian HIV-1 seropositive (index) partners and their seronegative (HESN) partners at baseline or during follow-up intervals.

	Index partners	HESNs		
Characteristics at baseline	*N* = 568	SCs (*n* = 240)	pHESNs (*n* = 328)	*p* value
Sex ratio (M/F)	1.1 (296/272)	0.6 (93/147)	1.2 (179/149)	0.0002
Age (years)				
Male (mean ± SD)	34.6±7.8	32.3±7.6	34.8±8.1	0.014
Female (mean ± SD)	27.4±5.9	26.3±6.2	28.6±7.2	0.004
**Characteristics in most recent 6 months of follow-up**				
Months of follow-up, Median (IQR)	25.5 (14–47)	18 (9–36)	31.5 (17–56)	0.0001
Behavioral and clinical risk score, Median (IQR)	1 (0–2)	1 (1–2)	1 (0–1)	<0.0001
HIV-1 viral load (log_10_) in index partner				
M, mean ± SD	4.93±0.74	NA	NA	
F, mean ± SD	4.44±0.84	NA	NA	

The *p* values in last column refer to comparisons between seroconverters (SCs) and persistent HIV-1 exposed seronegatives (pHESNs). Behavioral and clinical risk score includes combination of 1) genital ulcer/inflammation for either index, HESN, or both partners, 2) no circumcision of HESN male, 3) recent pregnancy in HESN female, and 4) sperm in vaginal fluid in HESN female in most recent 6 month of follow-up. F, female; IQR, interquartile range; M, male; NA, not applicable; SD, standard deviation of the mean.

### Selective testing of HLA markers with previously reported and newly detected associations

We first examined all HLA class I alleles or haplotypes implicated in HIV-1 infection or disease control in earlier studies of associations in Africans by both Cox proportional hazards and logistic regression analysis (**Table 2**, Group I). Two markers, A*68:02 and one or both of the virtually inseparable alleles in the B*42-C*17 haplotype, were associated with increased likelihood or rate of acquisition (*p*<0.05). Only the A*68:02 association withstood analysis for false discovery rate (FDR, q = 0.025 for Cox proportional hazards model and *q* = 0.050 for logistic regression model). The unfavorable effects of these markers here contrast starkly with their apparent protective roles previously reported: for A*68:02 against infection in another African population [26] and for the B*42-C*17 haplotype with lower viremia in both our and other cohorts [31], [32].

**Table 2:** Analyses of HLA class I variants previously or presently associated with acquisition or control of HIV-1 infection.

		SCs	pHESNs	Time to infection (Cox model)			Logistic regression (SCs vs. pHESNs)		
	Variants	*n* (%)	*n* (%)	RH (95% CI)	*p*	*q*	OR (95% CI)	*p*	*q*
**Group I**	A*68:02	51 (21.3)	38 (11.6)	1.67 (1.2–2.3)	0.001	0.025	2.06 (1.3–3.3)	0.002	0.050
	B*42-C*17	43 (17.9)	41 (12.5)	1.42 (1.0–2.0)	0.035	0.386	1.52 (1.0–2.4)	0.085	0.236
**Group II**	B*14	36 (15.0)	33 (10.0)	1.40 (1.0–2.0)	0.065	0.386	1.79 (1.0–3.1)	0.037	0.205
	B*44	17 (7.1)	41 (12.5)	0.70 (0.4–1.1)	0.161	0.391	0.52 (0.3–1.0)	0.034	0.205
	B*51	6 (2.5)	20 (6.1)	0.54 (0.2–1.2)	0.139	0.386	0.37 (0.2–1.0)	0.041	0.205
	B*51-C*16	5 (2.1)	18 (5.5)	0.50 (0.2–1.2)	0.121	0.398	0.33 (0.1–1.0)	0.036	0.205
**Group III**	A*01	5 (2.1)	17 (5.2)	0.45 (0.2–1.1)	0.075	0.398	0.37 (0.1–1.1)	0.055	0.229
	A*02	55 (22.9)	95 (29.0)	0.81 (0.6–1.1)	0.172	0.391	0.74 (0.5–1.1)	0.137	0.302
	A*23	60 (25.0)	79 (24.1)	1.08 (0.8–1.5)	0.612	0.805	1.02 (0.7–1.5)	0.927	0.927
	A*36	32 (13.3)	30 (9.2)	1.21 (0.8–1.8)	0.316	0.573	1.66 (1.0–2.8)	0.070	0.236
	A*68:01	6 (2.5)	14 (4.3)	0.76 (0.3–1.7)	0.514	0.756	0.66 (0.2–1.8)	0.411	0.642
	A*74:01	28 (11.7)	45 (13.7)	0.83 (0.6–1.2)	0.344	0.573	0.79 (0.5–1.3)	0.385	0.642
	B*18	22 (9.2)	29 (8.8)	1.06 (0.7–1.7)	0.792	0.923	1.06 (0.6–1.9)	0.853	0.927
	B*35	15 (6.3)	23 (7.0)	0.86 (0.5–1.4)	0.584	0.805	0.96 (0.4–2.1)	0.917	0.927
	B*53	55 (22.9)	57 (17.4)	1.01 (0.8–1.4)	0.955	0.992	1.39 (0.9–2.1)	0.126	0.302
	B*57	20 (8.3)	30 (9.2)	0.83 (0.5–1.3)	0.432	0.675	0.91 (0.5–1.7)	0.756	0.899
	B*5703	13 (5.4)	23 (7.0)	0.76 (0.4–1.3)	0.327	0.573	0.75 (0.4–1.6)	0.444	0.653
	B*5801	17 (7.1)	30 (9.2)	0.67 (0.4–1.1)	0.106	0.386	0.75 (0.4–1.4)	0.366	0.642
	B*5802	29 (12.1)	37 (11.3)	1.07 (0.7–1.6)	0.722	0.903	1.08 (0.6–1.8)	0.775	0.899
	B*81	16 (6.7)	19 (5.8)	1.00 (0.6–1.7)	0.992	0.992	1.10 (0.5–2.2)	0.791	0.899
	C*04	86 (35.8)	94 (28.7)	1.03 (0.8–1.4)	0.812	0.923	1.38 (1.0–2.0)	0.084	0.236
	C*18	27 (11.3)	41 (12.5)	0.81 (0.5–1.2)	0.305	0.573	0.85 (0.5–1.4)	0.539	0.749
	B*14−C*08	33 (13.8)	32 (9.8)	1.33 (0.9–1.9)	0.130	0.386	1.37 (0.8–2.3)	0.251	0.483
	B*44−C*04	12 (5.0)	18 (5.5)	0.95 (0.5–1.7)	0.866	0.941	0.89 (0.4–1.9)	0.762	0.899
	A*30+C*03	13 (5.4)	28 (8.5)	0.65 (0.4–1.1)	0.135	0.386	0.60 (0.3–1.2)	0.145	0.302

Analyses are based on 240 seroconverters (SCs) and 328 the persistent HIV-1 exposed seronegatives (pHESNs). Group I HLA variants include those associated with time to HIV-1 acquisition in both univariate and multivariable models (Table 3). Group II has variants that are rejected by multivariable models (adjusted *p*>0.050). Other variants under Group III have been highlighted in earlier studies relevant to heterosexual HIV-1 acquisition and/or immune control of infection. Variants highly relevant to Zambians are underlined. Age and gender are treated as covariates in all analyses. The *q* values correspond to false discovery rates.

Three additional markers involving *HLA-B* that have occasionally been implicated in studies of HIV-1 disease control in other populations but not in Zambians showed associations with acquisition of infection here with nominal *p*≤0.05: B*44 and either B*51 or the B*51-C*16 haplotype with lower risk (ORs = 0.52 and 0.37 or 0.33, respectively) and B*14 with higher risk (OR = 1.79) (**Table 2**, Group II). However, the statistical significance of those three markers exceeded the FDR threshold (all q≥0.2). No other variant previously associated with control of viremia [20] or transmission from the index partner [6] in Zambians and no other variant associated with acquisition in other cohorts (e.g., A*23 and B*18) [24], [25] appeared to influence the acquisition of HIV-1 infection in Zambian couples.

No other HLA class I allele that has been reproducibly found to influence HIV-1 infection or disease progression showed a corresponding association with HIV-1 acquisition in the full cohort of discordant Zambian couples (**Table 2**, Group III). Only in the subset of couples with female HESNs was there a suggestion that the African allele A*74:01, recently recognized in association with both lower VL and decreased likelihood of HIV-1 infection [20], [22], [28], [33], [34], was associated with slower rate of acquisition (adjusted univariate RH = 0.49, *p* = 0.04) (data not shown). The significance of this association was above the FDR threshold (*q* = 0.57). Although each comparison was adjusted for age and gender, this adjustment did not alter any of the associations.

Ten HLA class I supertypes were also analyzed in both Cox proportional hazards and logistic regression models. None of the 10 showed statistically significant effects on time to infection or on overall occurrence of infection by the end of study period (**Table S1**). A specifically designated portion of the A2 supertype, A02/A6802, was previously reported to retard acquisition [26]; however, here it was associated with accelerated acquisition (RH = 1.43, *p* = 0.013 and *q* = 0.165), and this effect was due entirely to the contribution of A*68:02, the dominant allele in this supertype (85 of 137 with the supertype).

### HLA-A*68:02 and B*42-C*17 as independent correlates of HIV-1 acquisition

**Figure 2:**
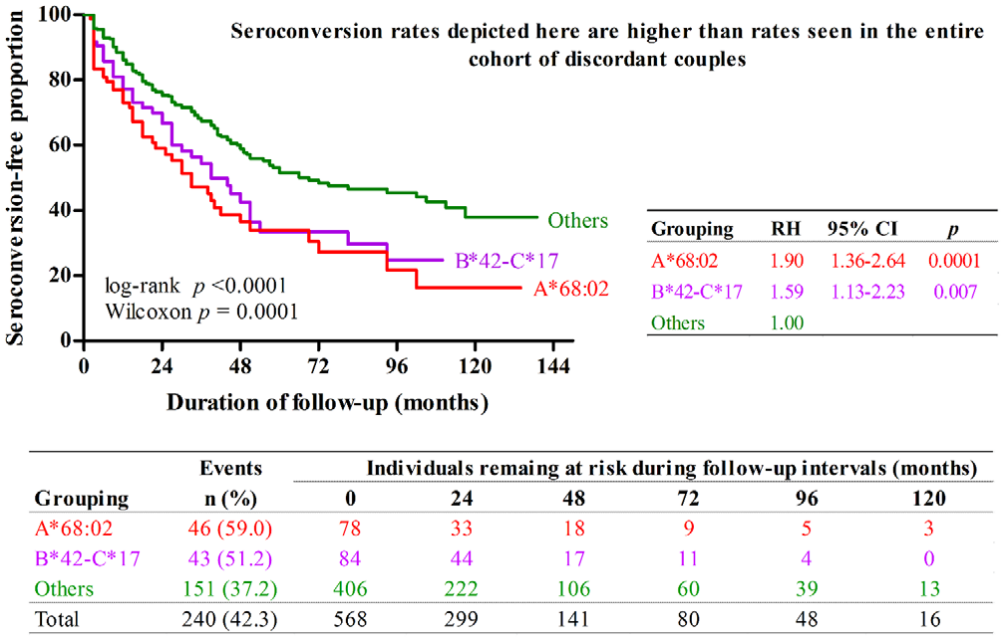
Kaplan-Meier plots for HIV-1 acquisition among 568 Zambian initial HESNs stratified by HLA-A*68:02 and B*42-C*17. Of the 11 individuals with both A*68:02 and B*42-C*17, five acquired HIV-1 at rates similar to those in carriers of B*42-C*17 alone; these 11 were treated as part of the B*42-C*17 group. Differences in acquisition-free time among the three HLA subgroups were tested for significance by the log-rank and Wilcoxon methods. Estimates of relative hazards (RH) of HIV-1 acquisition are based on Cox proportional hazards models. The numbers of subjects remaining at-risk at successive follow-up intervals are tabulated below the Kaplan-Meier plots. The seroconversion rate does not reflect the overall rate in the entire ZEHRP cohort, because couples in this study were selected for clinical and behavioral HIV-1 risk characteristics.

All alleles and haplotypes in Table 2 were further analyzed in multivariable Cox proportional hazards models with stepwise elimination. These models dismissed several probable or suspected HLA factors, including (i) B*57, B*81, and C*18 in index partners, (ii) B*14, B*44, B*81, B*51, and C*18 in HESNs (adjusted *p*≥0.05 for all). In the end, only A* 68:02 and B*42-C*17 in HESNs and A*36 in index partners retained statistical significance (adjusted relative hazards, RH = 1.76, 1.46 and 2.06; *p* = 0.0004, 0.025 and <0.0001, respectively) in a reduced model for comparing time to HIV-1 acquisition (**Table 3**, model I). The associations of all three of these HLA factors were independent of the behavioral, clinical and biological factors documented for the study population. The impact of these genetic markers persisted in a model including VL in index partners (Table 3, model II) as well as a model including both VL in index partners and risk score in couples (RH = 1.57, 1.55 and 1.78; *p* = 0.007, 0.013 and 0.001) (**Table 3**, model III). Of the 11 initial HESNs who had both A*68:02 and B*42-C*17, five seroconverted at a rate similar to that seen with B*42-C*17 alone. The infection-free time among initial HESNs bearing A*68:02 (median = 33 months, 95% confidence interval, CI = 21–45 months) and those bearing B*42-C*17 (median = 39 months, 95% CI = 27–51 months) contrasted with that among other initial HESNs bearing neither variant (median = 69 months, 95% CI = 51–111 months). During the 11-year study interval, 59.0% of A*68:02-positive and 51.2% of B*42-C*17-positive initial HESNs became infected. HESNs with either of those two markers showed significantly more rapid rates of seroconversion than the remaining HESNs (*p*<0.001 by log-rank and Wilcoxon tests) (**Figure 2**).

**Table 3:** Association of HLA class I variants with HIV-1 acquisition in multivariable models including only genetic variables or behavioral and biologic risk indicators.

	All couples			MTF couples			FTM couples		
	RH	95% CI	*P*	RH	95% CI	*P*	RH	95% CI	*P*
**Model I**									
A*68:02 in HESNs	1.76	1.3–2.4	0.0004	1.75	1.2–2.5	0.004	1.47	0.8–2.6	0.185
B*42−C*17 in HESNs	1.46	1.1–2.0	0.025	1.49	1.0–2.3	0.068	1.54	0.9–2.6	0.116
A*36 in index partners	2.06	1.5–2.9	<0.0001	1.52	0.9–2.6	0.115	3.00	1.9–4.9	<0.0001
**Model II**									
A*68:02 in HESNs	1.78	1.3–2.5	0.0004	1.80	1.2–2.7	0.003	1.60	0.9–2.8	0.109
B*42−C*17 in HESNs	1.50	1.1–2.1	0.020	1.53	1.0–2.4	0.066	1.68	1.0–2.9	0.059
A*36 in index partners	1.78	1.3–2.5	0.001	1.39	0.8–2.4	0.226	2.39	1.5–3.9	0.001
VL in index partners	1.77	1.5–2.2	<0.0001	1.48	1.1–1.9	0.005	1.96	1.4–2.7	<0.0001
**Model III**									
A*68:02 in HESNs	1.57	1.1–2.2	0.007	1.38	1.0–2.1	0.122	1.48	0.8–2.7	0.180
B*42−C*17 in HESNs	1.55	1.1–2.2	0.013	1.75	1.1–2.8	0.017	1.38	0.8–2.4	0.242
A*36 in index partners	1.78	1.3–2.5	0.001	1.53	0.9–2.6	0.122	2.29	1.4–3.7	0.001
VL in index partners	1.81	1.5–2.2	<0.0001	1.38	1.1–1.8	0.018	1.83	1.3–2.5	0.0001
Risk score									
1	1.39	1.0–2.0	0.082	2.01	1.3–3.1	0.001	2.48	0.7–8.2	0.136
2	3.58	2.5–5.2	<0.0001	4.73	2.9–7.7	<0.0001	7.62	2.3–24.9	0.001
≥3	5.42	3.4–8.7	<0.0001	4.21	2.2–7.9	<0.0001	16.70	4.7–58.8	<0.0001

Abbreviations are defined in Table 1. Associations are tested using Cox proportional hazards models. In model III, each level of positive risk score is compared to the level in the reference group with a neutral score (zero).

### Analysis of GUI as a potential confounder

Among HESN partners, 23% had GUI during the 6 months prior to transmission or the end of follow-up. However, GUI was not associated with HLA class I variants of interest (**Table S2**).

## Discussion

In a comprehensive analysis of a cohort of HIV-1-serodiscordant couples including the largest known set of transmission pairs, none of the *HLA-B* alleles most widely reported to influence viremia, CD4 count or disease progression in subtype C infection were associated with occurrence or rate of acquisition of HIV-1 infection in the HESN partners. Specifically, although B*13:02, B*18, B*45:01, B*57:01-03, B*58:01, B*58:02, and B*81:01 have all been implicated as determinants of VL in sub-Saharan African populations [6], [19], [33], they had no corresponding effect on acquisition of HIV-1 subtype C infection in Zambians.

One *HLA-B* allele (B*42:01) that has shown some consistency in its association with better control of viremia [32], [33], [35]had a clearly unfavorable effect on acquisition (**Table 1**). The B*42-C*17 haplotype is carried in 18.8% of Zambians. Strong linkage disequilibrium (LD) between B*42 and C*17 in our study population (*r* = 0.97) [20] precluded separate analysis of either B*42 or C*17 alone. However, these two alleles occur frequently in the A*30-C*17-B*42 haplotype, and the favorable effect of A*30 seen earlier [20] makes it an unlikely contributor to the acceleration of infection seen here with the B–C haplotype.

Additional *HLA-B* alleles (B*14, B*44 and B*51) were differentially distributed between SCs and pHESNs; however, their impact diminished with more comprehensive analyses. Relatively slow disease progression has been documented for B*51 in individuals infected largely with subtype B [9], [17], but that pattern has not extended to other populations, and association with lower susceptibility to infection has been inconsistent [36]. At the population level, mutations in HIV-1 induced by B*51 may account for viral evolution to fixation in infected individuals, but it is difficult to predict how such changes in the virus might affect acquisition in HESNs [37]. Findings with B*14 and B*44 have been even less consistent across ethnic and viral subtype boundaries [38], [39]. The contributions of these alleles to susceptibility are unclear and appear to be minor. Taken as a whole, such strikingly disparate effects of *HLA-B* alleles on the two outcome measures (control of VL and susceptibility to infection) in the same cohort imply that if *HLA-B* alleles play any substantial role in modulating resistance or susceptibility to infection, the convincingly documented mechanism by which those alleles mediate CTL response to specific viral epitopes during the course of subtype C infection is unlikely to represent the dominant mechanism regulating heterosexual acquisition.

As for *HLA-A* alleles, only the relatively recently investigated African HLA-A*74:01 has shown a comparably favorable effect on both VL in HIV-infected individuals [20], [22], [33] and acquisition among Tanzanians [28] as well as in HESN Zambian women in our study. Its linkage disequilibrium (LD) with B*57 could not explain the association with slower acquisition in Zambian women because the latter allele showed no such effect in the absence of A*74:01. This *HLA-A* allele does not show predominant LD with any particular *HLA-C* allele, but its pattern of LD in the class I region raises the possibility that it tags a variant involved in one of the natural killer cell pathways. The findings for A*74:01 across populations of African ancestry merit further investigation.

The strongest association seen among Zambian HESNs with any class I allele was between HLA-A*68:02 and accelerated HIV-1 acquisition. The relationship was opposite to the protective association previously reported with this allele as part of the A02 supertype in Kenyan commercial sex workers heavily exposed to HIV-1 subtype A [23], [26]. A*68:02 also appeared to be associated with relative resistance to HIV-1 subtype B infection among men of European ancestry [40]. Differential effects in the setting of different viral subtypes could explain these discrepant associations. A haplotype effect would be another possible reason for the population-specific findings. However, in Zambians A*68:02 is found most frequently in the A*68:02-C*03-B*15 haplotype–but in only 5% of HESNs and not in tight LD. Regardless of the population-specific findings, if there is a biologic basis for the association of A*68:02 with susceptibility to subtype C infection in Zambians, a similarly unfavorable relationship has not been reported for viremia or disease control with any HIV-1 subtype. Conversely, A*36 in HESN Zambians did not contribute to the acquisition of infection, even though this allele was associated with both higher VL in their index partners and accelerated viral transmission from them to their HESN partners [6], [20]. Thus, for both of these *HLA-A* alleles our data suggest that the mechanisms underlying immune control of viremia and acquisition of HIV-1 infection are indeed distinct [6], [21].

Our earlier analysis of killer immunoglobulin-like receptor (KIR) genes has revealed some evidence that genetic associations with HIV-1 transmission may operate indirectly through prominent cofactors (e.g., coinfections). Specifically, KIR2D4*001 as an unfavorable marker in the Zambian cohort had dual associations with HIV-1 transmission by index partners and genital ulcer in index partners [41]. Genital ulcers and inflammation (GUI) also represent a prominent cofactor for HIV-1 acquisition by HESNs, but the HLA class I variants highlighted here had no appreciable impact on GUI; that is, GUI could not have significantly confounded the observed HLA effects on HIV-1 acquisition.

The frequency of *HLA-B* supertypes has been inversely related to the degree of HIV-1 disease control in infected individuals of European and of African ancestry [22], [42], [43]. However, our analyses excluded any substantial contribution by either *HLA-A* or *HLA-B* supertypes to HIV-1 acquisition in Zambians. As with the individual alleles, our data imply that the differences in peptide-binding patterns captured by supertype clustering do not influence acquisition of HIV-1 infection in the way they do HIV-1 disease control.

These findings could have important implications for the design of CTL-based vaccines. The evidence for the occurrence of CTL responses in HESNs has been inconsistent [44], [45], [46]. However, even if it could be conclusively shown that HLA allele-specific CTL responses differentially promote or retard acquisition of infection, the profile of the genetic polymorphisms that enhance or impede that process appears to be so different from the profile of those controlling post-seroconversion immunologic events that the strategies for designing CTL-based prophylactic and therapeutic vaccines would almost surely have to diverge along those lines [30].

In summary, our data indicate that variation according to individual *HLA-A*, *-B* and *–C* alleles in HESNs and, by implication, whatever differential CTL responses may be mediated by those different alleles, fail to explain much if any variation in resistance to infection among Zambian partners exposed to HIV-1 subtype C. Of course, these disparate results for individual alleles do not preclude possible contributions of class I molecules by any of several alternative mechanisms. These alternatives include differential interaction of class I proteins with non-classical class I molecules [47], [48] receptors in NK cell pathways [49], [50], chaperones [51], T-cell receptors [52], and immunoglobulin-like transcript 4 molecules [53]. Hopefully, the context of cohabiting serodiscordant couples will afford further opportunity to elucidate the distinctive roles of HLA class I polymorphism in acquisition of HIV-1 infection and in disease control.

## Materials and Methods

### Ethics Statement

This study followed the human experimentation guidelines of the United States Department of Health and Human Services, and all enrolled patients provided written informed consent. The work presented here was further approved by Institutional Review Boards at University of Alabama at Birmingham, on a yearly basis.

### Study Population

From 1995 through 2006 in the Zambia-Emory HIV Research Project (ZEHRP) we prospectively evaluated cohabiting HIV-1 serodiscordant couples consisting of a seropositive index partner and an HESN partner. The procedures for participant recruitment, counseling, quarterly follow-up, clinical examination (for genital ulcer/inflammation) and laboratory testing (for HIV-1 serology and viral load) have been described elsewhere [5], [54], [55], [56]. All couples whose HESN partner acquired virologically linked HIV-1 from the known index partner during follow-up were included in this study. Non-transmitting couples were selected on the basis of behavioral or clinical measures of sexual exposure. To concentrate on the level of increased risk experienced by each couple during the most recent six months of follow-up, we cumulated known predictors of risk for heterosexual transmission present at the two most recent quarterly visits [circumcision, genital ulcer/inflammation (GUI) by history or examination, pregnancy, and sperm in vaginal fluid] in a composite risk score. Although average risk scores were lower in the non-transmitting couples available for study than in transmitting couples, we disproportionally sampled non-transmitting couples with greater exposure to risk predictors. For transmitting couples, viruses detected in seroconverters had to be similar to those present in their cohabiting index partners by phylogenetic analysis of subgenomic HIV-1 sequences corresponding to *gag*, *env* and the long terminal repeat regions [5], [57]. Couples with unlinked or ambiguous viruses or inadequate follow-up time (<9 months) or missing information were excluded. Through December 2006, 568 couples became eligible for analyses (**Figure 1**).

### HLA class I typing

Methods for HLA class I genotyping have been described elsewhere [6], [20]. In brief, genomic DNA extracted from buffy coats or whole blood was used for PCR with sequence-specific primers (SSP) (Dynal/Invitrogen, Brown Deer, WI), sequence-specific oligonucleotide (SSO) probe hybridization (Innogenetics, Alpharetta, GA), and sequencing-based typing (SBT) (Abbott Molecular, Inc., Des Plaines, IL). Most HLA class I alleles were defined to 4-digit specificities. HLA haplotypes and supertypes were assigned using the expectation-maximization (EM) algorithm in SAS Genetics (SAS Institute, Cary, NC) [6], [20].

### Supertype assignment

HLA class I supertypes were assigned according to previous classification and a recent update [22], [43], [58] designating four *HLA-A* supertypes (A01, A02, A03 and A24) and six *HLA-B* supertypes (B07, B08, B27, B44, B58 and B62). For analytic purposes here A*3001 and A*2902 were left unclassified along with other unclassified supertypes according to the previous framework [58]. For comparison with previous studies we also clustered several related *HLA-A* alleles into a supertype designated A02/6802 [40].

### Statistical Analysis

Earlier analyses of the Zambian cohort have addressed various aspects of HIV-1 transmission and VL [5], [6], [20], [54], [55], [56]. Here, we focused on deciphering the role of HLA class I variants [2- and 4-digit allele levels (**Figure 1b**), haplotypes, and supertypes] in HIV-1 acquisition among Zambian HESNs. Statistical software packages in SAS 9.2 with SAS/Genetics (SAS Institute Inc., Cary, NC) were used for all analyses. We first assessed non-genetic factors, including age, gender, risk score, direction of HIV-1 transmission (male-to-female and female-to-male) and index partner viral load (VL) (**Table 1**). Distribution of class I alleles were evaluated for a) Hardy-Weinberg equilibrium (HWE), b) association with time to HIV-1 acquisition (Cox proportional hazards models and Kaplan-Meier plots), c) association with HIV-1 infection status at the end of follow-up (logistic regression models). Kaplan-Meier plots illustrate differences in transmission associated with specific genetic markers; because non-transmitting couples with relatively higher frequencies of risk predictors were selectively included, these transmission rates do not reflect rates in the entire prospectively observed discordant couple population in Zambia. The overall annual HIV-1 seroincidence (7–9/100 PY) represents a one-half to two-thirds reduction in transmission following the introduction of joint testing and counseling [1].

Multivariable analysis highlighted genetic and non-genetic factors that showed independent associations with HIV-1 acquisition, after stepwise elimination (*p*>0.05) of probable or suspected HLA factors. In light of the multiple comparisons performed, we also calculated a false discovery rate *q* value for all HLA variants formally tested in univariate models (**Table 2**) [59]. Alleles with *q*<0.2 received further consideration, as suggested by earlier work [60].

## Supporting Information

Table S1 **Lack of association between HLA class I supertypes and HIV-1 acquisition among 568 Zambians cohabiting with HIV-1 seropositive partners.**
(DOC)

Table S2 **Lack of association between HLA class I variants and genital ulcer/inflammation in 568 Zambians who were HIV-1 seronegative at enrollment.**
(DOC)
