# Supplementary material for: Disparate Associations of HLA Class I Markers with HIV-1 Acquisition and Control of Viremia in an African Population
Source: PLoS One. 2011 Aug 17;6(8):e23469. doi: 10.1371/journal.pone.0023469 (PMC3157381; doi:10.1371/journal.pone.0023469)
Supplement: Table S1 — Lack of association between HLA class I supertypes and HIV-1 acquisition among 568 Zambians cohabiting with HIV-1 seropositive partners. (DOC) [file pone.0023469.s001.doc]

**Table S1.** Lack of association between HLA class I supertypes and HIV-1 acquisition among 568 Zambians cohabiting with HIV-1 seropositive partners.

| **HLA supertypes** | **Total subjects** | **SCs** | **pHESNs** | **Time to HIV-1 infection (Cox models)** | | | **SCs vs. pHESNs by logistic regression** | | |
| --- | --- | --- | --- | --- | --- | --- | --- | --- | --- |
|  |  | ***n* (%)** | ***n* (%)** | **RH (95% CI)** | ***p*** | ***q*** | **OR (95% CI)** | ***p*** | ***q*** |
| A01 | 22 | 5 (2.1) | 17 (5.2) | 0.47 (0.2-1.1) | 0.091 | 0.504 | 0.39 (0.1-1.1) | 0.068 | 0.361 |
| A02 | 150 | 55 (22.9) | 95 (29.0) | 0.81 (0.6-1.1) | 0.159 | 0.508 | 0.73 (0.5-1.1) | 0.107 | 0.361 |
| A03 | 228 | 88 (36.7) | 140 (42.7) | 0.81 (0.6-1.1) | 0.116 | 0.504 | 0.78 (0.6-1.1) | 0.149 | 0.361 |
| A24 | 149 | 64 (26.7) | 85 (25.9) | 1.05 (0.8-1.4) | 0.756 | 0.941 | 1.04 (0.7-1.5) | 0.840 | 0.910 |
| A02/A6802 | 137 | 67 (27.9) | 70 (21.3) | 1.43 (1.1-1.9) | 0.013 | 0.165 | 1.43 (1.0-2.1) | 0.071 | 0.361 |
| Other A’s | 175 | 81 (33.8) | 94 (28.7) | 1.17 (0.9-1.5) | 0.259 | 0.508 | 1.27 (0.9-1.8) | 0.195 | 0.361 |
|  |  |  |  |  |  |  |  |  |  |
| B07 | 315 | 141 (58.8) | 174 (53.1) | 1.13 (0.9-1.5) | 0.352 | 0.508 | 1.26 (0.9-1.8) | 0.177 | 0.361 |
| B08 | 42 | 16 (6.7) | 26 (7.9) | 1.02 (0.6-1.7) | 0.941 | 0.941 | 0.83 (0.4-1.6) | 0.571 | 0.743 |
| B27 | 229 | 97 (40.2) | 132 (40.2) | 1.02 (0.8-1.3) | 0.886 | 0.941 | 1.01 (0.7-1.4) | 0.967 | 0.967 |
| B44 | 212 | 87 (36.3) | 125 (38.1) | 0.97 (0.7-1.3) | 0.811 | 0.941 | 0.92 (0.7-1.3) | 0.651 | 0.770 |
| B58 | 169 | 67 (27.9) | 102 (31.1) | 0.83 (0.6-1.1) | 0.203 | 0.508 | 0.86 (0.6-1.2) | 0.413 | 0.596 |
| B62 | 24 | 7 (2.9) | 17 (5.2) | 0.66 (0.3-1.1) | 0.279 | 0.508 | 0.55 (0.2-1.3) | 0.191 | 0.361 |
| Other B’s | 3 | 2 (0.8) | 1 (0.3) | 2.01 (0.5-8.1) | 0.327 | 0.508 | 2.75 (0.3-30.5) | 0.410 | 0.596 |

SCs and pHESNs are as defined in Table 1 and Figure 1. The A02/6802 supertype consists of alleles like A*02:02, A*02:05, A*02:14 and A*68:02 (MacDonald et al., *J Infect Dis* 2000; 181: 1581-1589); its weak association with HIV-1 acquisition can be explained by A*68:02 alone (see Table 2). The unclassified *HLA-A* alleles mostly capture A*29:02 and A*30:01 (Sidney et al., *BMC Immunol* 2008; 9:1), while unclassified *HLA-B* alleles (Sidney et al., *BMC Immunol* 2008; 9:1) are rarely seen in the study population. RH, relative hazards; OR, odds ratio; CI, confidence interval; *q*, false discovery probability.
