# Supplementary material for: Disparate Associations of HLA Class I Markers with HIV-1 Acquisition and Control of Viremia in an African Population
Source: PLoS One. 2011 Aug 17;6(8):e23469. doi: 10.1371/journal.pone.0023469 (PMC3157381; doi:10.1371/journal.pone.0023469)
Supplement: Table S2 — Lack of association between HLA class I variants and genital ulcer/inflammation in 568 Zambians who were HIV-1 seronegative at enrollment. (DOC) [file pone.0023469.s002.doc]

**Table S2.** Lack of association between HLA class I variants and genital ulcer/inflammation in 568 Zambians who were HIV-1 seronegative at enrollment.

| **HLA variants** | | **Unadjusted models** | | | **Adjusted models** | | |
| --- | --- | --- | --- | --- | --- | --- | --- |
|  |  | **OR (95% CI)** | ***p*** | ***q*** | **OR (95% CI)** | ***p*** | ***q*** |
| **Group I** | A*68:02 | 1.35 (0.8-2.3) | 0.265 | 0.704 | 1.33 (0.8-2.3) | 0.297 | 0.792 |
|  | B*42-C*17 | 1.19 (0.7-2.0) | 0.529 | 0.704 | 1.16 (0.7-2.0) | 0.599 | 0.825 |
|  |  |  |  |  |  |  |  |
| **Group II** | B*14 | 1.33 (0.7-2.4) | 0.333 | 0.704 | 1.16 (0.6-2.1) | 0.627 | 0.825 |
|  | B*44 | 0.50 (0.2-1.1) | 0.078 | 0.704 | 0.54 (0.2-1.1) | 0.089 | 0.792 |
|  | B*51 | 0.81 (0.3-2.2) | 0.679 | 0.793 | 0.81 (0.3-2.3) | 0.679 | 0.849 |
|  | B*51-C*16 | 0.72 (0.2-2.2) | 0.563 | 0.704 | 0.69 (0.2-2.1) | 0.521 | 0.825 |
|  |  |  |  |  |  |  |  |
| **Group III** | A*01 | 0.54 (0.2-1.9) | 0.326 | 0.704 | 0.50 (0.1-1.7) | 0.276 | 0.792 |
|  | A*02 | 0.87 (0.5-1.4) | 0.541 | 0.704 | 0.89 (0.6-1.4) | 0.624 | 0.825 |
|  | A*23 | 1.36 (0.9-2.1) | 0.176 | 0.704 | 1.34 (0.9-2.1) | 0.201 | 0.792 |
|  | A*36 | 1.29 (0.7-2.4) | 0.402 | 0.704 | 1.30 (0.7-2.4) | 0.412 | 0.792 |
|  | A*68:01 | 0.36 (0.1-1.6) | 0.170 | 0.704 | 0.38 (0.1-1.7) | 0.205 | 0.792 |
|  | A*74:01 | 0.70 (0.4-1.3) | 0.281 | 0.704 | 0.68 (0.4-1.3) | 0.243 | 0.792 |
|  | B*18 | 0.78 (0.4-1.6) | 0.497 | 0.704 | 0.81 (0.4-1.7) | 0.567 | 0.825 |
|  | B*35 | 0.96 (0.4-2.1) | 0.917 | 0.955 | 0.96 (0.4-2.1) | 0.917 | 0.921 |
|  | B*53 | 1.33 (0.8-2.1) | 0.243 | 0.704 | 1.26 (0.8-2.0) | 0.351 | 0.792 |
|  | B*57 | 0.72 (0.3-1.5) | 0.383 | 0.704 | 0.71 (0.3-1.5) | 0.39 | 0.792 |
|  | B*5703 | 0.50 (0.2-1.3) | 0.157 | 0.704 | 0.49 (0.1-1.3) | 0.159 | 0.792 |
|  | B*5801 | 0.60 (0.3-1.4) | 0.229 | 0.704 | 0.60 (0.3-1.4) | 0.237 | 0.792 |
|  | B*5802 | 1.06 (0.6-2.0) | 0.845 | 0.919 | 1.07 (0.6-2.0) | 0.844 | 0.917 |
|  | B*81 | 0.65 (0.3-1.6) | 0.348 | 0.704 | 0.63 (0.3-1.5) | 0.299 | 0.792 |
|  | C*04 | 1.09 (0.7-1.6) | 0.698 | 0.793 | 1.05 (0.7-1.6) | 0.833 | 0.917 |
|  | C*18 | 0.99 (0.5-1.8) | 0.976 | 0.976 | 0.97 (0.5-1.8) | 0.921 | 0.921 |
|  | B*14-C*08 | 1.20 (0.7-2.2) | 0.502 | 0.704 | 1.09 (0.6-2.0) | 0.785 | 0.917 |
|  | B*44-C*04 | 0.70 (0.3-1.9) | 0.469 | 0.704 | 0.71 (0.3-1.9) | 0.501 | 0.825 |
|  | A*30+C*03 | 0.64 (0.3-1.5) | 0.296 | 0.704 | 0.65 (0.3-1.5) | 0.325 | 0.792 |

HLA variants correspond to those listed in Table 2. B*44 shows a slight trend for association, before and after statistical adjustments for gender and baseline age. OR, odds ratio; CI, confidence interval; *q*, false discovery probability.
